# Recent advances in triple negative breast cancer: the immunotherapy era

**DOI:** 10.1186/s12916-019-1326-5

**Published:** 2019-05-09

**Authors:** Antonio Marra, Giulia Viale, Giuseppe Curigliano

**Affiliations:** 1 Division of Early Drug Development for Innovative Therapy, European Institute of Oncology (IEO), IRCCS, Milan, Italy; 2 Department of Oncology and Haematology, University of Milano, Milano, Italy

**Keywords:** Triple-negative breast cancer, Immunotherapy, PD-L1, Biomarkers, Checkpoint inhibitors

## Abstract

**Background:**

Several accomplishments have been achieved in triple-negative breast cancer (TNBC) research over the last year. The phase III IMpassion130 trial comparing chemotherapy plus atezolizumab versus chemotherapy plus placebo brought breast cancer into the immunotherapy era. Nevertheless, despite encouraging results being obtained in this trial, many open questions remain.

**Main body:**

A positive overall survival outcome was achieved only in PD-L1^+^ TNBC patients, suggesting a need to enrich the patient population more likely to benefit from an immunotherapeutic approach. Moreover, it remains unknown whether single-agent immunotherapy might be a good option for some patients. In this context, the discovery and implementation of novel and appropriate biomarkers are required. Focusing on the early onset of TNBC, neoadjuvant trials could represent excellent in vivo platforms to test immunotherapy agents and their potential combinations, allowing the performance of translational studies for biomarker implementation and improved patient selection.

**Conclusion:**

The aim of our review is to present recent advances in TNBC treatment and to discuss open issues in order to better define potential future directions for immunotherapy in TNBC.

## Background

Triple-negative breast cancer (TNBC) accounts for approximately 15–20% of all breast carcinomas and is associated with earlier age of onset, aggressive clinical course, and dismal prognosis compared to hormone receptor- and HER2-positive breast carcinomas [1]. Given the lack of effective treatments in this subtype of breast cancer, several efforts have been conducted in recent years to increase the therapeutic opportunities for TNBC patients.

Over the last 10 years, considerable evidence has highlighted the primary role of the immune system in influencing the disease course of TNBC. The presence of tumor-infiltrating lymphocytes (TILs), assessed by means of immunohistochemistry staining, is widely recognized as a predictor of good prognosis in both adjuvant and neoadjuvant settings of TNBC [2–5]. In addition, a deeper characterization of immune infiltrates, including the presence of a high number of cytotoxic (CD8^+^) TILs or a high CD8^+^/FOXP3^+^ ratio, is able to define TNBC patients with a better prognosis following neoadjuvant chemotherapy [6]. Along with the presence of TILs, the expression of immune evasion molecules in the tumor microenvironment, such as programmed death-ligand 1 (PD-L1), has also been shown to influence TNBC prognosis [7–10]. These data, together with the development of new therapeutic agents directed against immune checkpoint molecules, such as anti-PD-1 and anti-PD-L1 monoclonal antibodies, provide the rationale for the assessment of immunotherapeutic approaches in TNBC patients.

New and relevant evidence on the implementation of immune checkpoint-based treatments in TNBC has emerged over the last year, with the results of the IMpassion130 trial bringing breast cancer into the immunotherapy era. Schmid et al. [11] demonstrated a substantial overall survival (OS) benefit in patients with PD-L1-positive (PD-L1^+^) metastatic or inoperable locally advanced TNBC through the addition of the anti-PD-L1 agent atezolizumab to first-line chemotherapy with nab-paclitaxel. Approximately 60% of the enrolled patients (451 for each treatment arm) experienced relapse after prior adjuvant/neoadjuvant treatment, while 37% presented with de novo stage IV disease. Additionally, approximately 41% of patients in the intent-to-treat (ITT) population had PD-L1^+^ disease. At a median follow-up of 12.9 months, the median progression-free survival (PFS) in the ITT population was significantly improved following the addition of atezolizumab as compared to chemotherapy alone (7.2 vs. 5.5 months); further, among the PD-L1^+^ population, the respective PFS benefit was more pronounced (7.5 vs. 5.0 months). An interim OS analysis showed that the OS difference was not statistically significant in the ITT population (median OS, 21.3 months (atezolizumab + chemotherapy) vs. 17.6 months (chemotherapy alone)). However, a statistically significant median OS increase of 9.5 months was observed with the addition of atezolizumab in the PD-L1^+^ population (25.0 vs. 15.5 months). Moreover, the objective response rate (ORR) was numerically higher following the addition of atezolizumab in both the ITT population (56% vs. 46%) and the PD-L1^+^ population (59% vs. 43%), and more complete responses were observed with atezolizumab than without (ITT, 7% vs. 2%; PD-L1^+^ population, 10% vs. 1%).

The above data thus prompt the question of whether immunotherapy would truly be transformative for metastatic TNBC. Despite the encouraging results, various questions have arisen from the IMpassion130 trial, including how to appropriately assess tumors for PD-L1 expression given the benefits derived from atezolizumab treatment in this patient group, which companion diagnostic is the most suitable, whether PD-L1 should be tested on immune cells or tumor cells, whether nab-paclitaxel is the ideal chemotherapy partner for immune checkpoint inhibitors (ICIs), whether an atezolizumab monotherapy arm that might present a good option for a selected subset of patients was missed, or assessing what can be learnt from the neoadjuvant setting. The aim of the present review is to discuss these open questions in order to define potential future directions for immunotherapy in TNBC.

## How to enrich the TNBC population: PD-L1 and emerging biomarkers

The positive results obtained in the PD-L1^+^ subgroup in the IMpassion130 trial suggest that there is a need to enrich the study population. However, PD-L1 is not the ideal biomarker to select patients for anti-PD1/PD-L1 therapies, as demonstrated in other cancers. Indeed, only a subset of patients with PD-L1^+^ tumors derives a real clinical benefit from immunotherapeutic treatment, while these therapies can determine clinical and radiological responses also in PD-L1^−^ cancers. Considering multiple parameters which take into consideration both tumor- and patient-related characteristics, a comprehensive view of immunotherapy in cancer treatment has been proposed and included in the framework of the cancer immunogram [12]. Given the potential toxicities of immunotherapy and the highly variable response across tumor types, as well as the significant economic burden of immunotherapeutic agents, the identification and implementation of novel biomarkers that can predict immunotherapeutic response are urgently required.

### PD-L1 expression

PD-L1 expression on tumor cells and/or immune infiltrate cells is considered a useful biomarker of treatment response following anti-PD-1 or anti-PD-L1 therapies [13, 14]. PD-L1 assessment is indeed used as a predictive biomarker in other tumor types such as non-small cell lung cancer [15]. Nevertheless, considerable gaps remain in our knowledge of the technical aspects of this test, including the biologic implications and associations of PD-L1 expression, the dynamic changes in expression, the heterogeneity in expression on tumor cells and on immune cells, and prognostic and/or predictive implications [16].

PD-L1 expression in TNBC has been shown to range from 40 to 65% as tested, in most cases, in immune cells [7–10, 17]. In the IMpassion130 study, a PD-L1 expression above 1% in immune cells was used to define the PD-L1^+^ group [11]. Interestingly, the majority of patients testing as PD-L1^+^ in immune cell tumors also had a positive PD-L1 expression on tumor cells [18]. In the Impassion130 biomarker subgroup analysis [18], PD-L1 expression on immune cells was positively correlated with CD8^+^ T cell number, and both factors were conjointly associated with increased PFS and OS. However, the best method to test PD-L1 expression in breast cancer should be harmonized. Since patients with PD-L1^−^ tumors may still obtain a clinical response with ICIs, PD-L1 expression should be used only to define a subgroup of patients expected to achieve greater benefit from ICIs rather than to exclude patients from treatment [19]. Nevertheless, this is certainly a provocative statement, underlined by the fact that more mature data from IMpassion130 and other trials with pembrolizumab in the same setting are still awaited in order to be able to draw final conclusions on this issue (Table 1). Therefore, a variety of potential biomarkers are now being assessed to predict immunotherapeutic efficacy in breast cancer beyond PD-L1 expression, including gene signatures, TILs, tumor mutational burden (TMB), microsatellite instability (MSI), and mismatch repair (MMR) deficiency.

**Table 1:** Ongoing phase II/III randomized immunotherapy trials in triple-negative breast cancer

Setting	Trial	Phase	Regimen	Patients	Status
Neoadjuvant	NCT03639948 (NeoPACT)	II	Carboplatin + docetaxel + pembrolizumab	100	R
	NCT03289819	II	Pembrolizumab + Nab-paclitaxel ➔ pembrolizumab + epirubicin and cyclophosphamide	50	R
	NCT03356860 (B-IMMUNE)	II	Paclitaxel + epirubicin + cyclophosphamide ± durvalumab	57	R
	NCT02685059 (GeparNuevo)	II	Epirubicin + nab-paclitaxel + cyclophosphamide ± durvalumab	174	ANR
Neoadjuvant/Adjuvant	NCT03036488 (KEYNOTE-522)	III	Carboplatin + paclitaxel + (anthracycline) + cyclophosphamide ± pembrolizumab➔ pembrolizumab	1174	ANR
	NCT03281954	III	Doxorubicin + cyclophosphamide + paclitaxel + carboplatin ± atezolizumab ➔ atezolizumab	1520	R
	NCT03197935 (IMpassion031)	III	Doxorubicin + cyclophosphamide + nab-paclitaxel ± atezolizumab ➔ atezolizumab	204	ANR
Adjuvant only for patients with residual disease after neoadjuvant chemotherapy	NCT02954874	III	Pembrolizumab vs. observation	1000	R
	NCT03756298	II	Capecitabine ± atezolizumab	284	R
Adjuvant	NCT03498716 (IMpassion030)	III	Paclitaxel ➔ dose-dense doxorubicin/epirubicin + cyclophosphamide ± atezolizumab	2300	R
	NCT02926196 (A-Brave)	III	Avelumab vs. observation	335	R
Locally advanced or metastatic TNBC	NCT02768701	II	Cyclophosphamide + pembrolizumab	40	ANR
	NCT03121352	II	Carboplatin, nab-paclitaxel and pembrolizumab	30	R
	NCT02819518 (KEYNOTE-355)	III	Abraxane or paclitaxel or carboplatin/gemcitabine ± pembrolizumab	858	ANR
	NCT02555657 (KEYNOTE-119)	III	Capecitabine, eribulin, gemcitabine, or vinorelbine as TPC vs. pembrolizumab	600	ANR
	NCT03644589	II	Cisplatin + pembrolizumab	60	NYR
	NCT02755272	II	Carboplatin + gemcitabine ± pembrolizumab	87	R
	NCT02447003 (KEYNOTE-086)	II	Pembrolizumab monotherapy	285	ANR
	NCT03125902 (IMpassion131)	III	Paclitaxel ± atezolizumab	540	R
	NCT03164993 (ALICE)	II	Pegylated liposomal doxorubicin + cyclophosphamide ± atezolizumab	75	R
	NCT03206203	II	Carboplatin + gemcitabine	185	R
	NCT03606967	II	Nab-paclitaxel + durvalumab ± neoantigen vaccine	70	NYR
	NCT03616886 (SYNERGY)	II	Paclitaxel, carboplatin, durvalumab ± oleclumab	171	R
	NCT03371017 (IMpassion132) (early recurrent)	III	Carboplatin + gemcitabine or capecitabine ± atezolizumab	350	R
	NCT03167619 (DORA)	II	Durvalumab + olaparib	60	R

*ANR* active, not recruiting, *NYR* not yet recruiting, *TPC* therapy per physician’s choice, *R* recruiting

### Tumor-infiltrating lymphocytes (TILs)

TILs are a well-known prognostic factor in early-stage TNBC, positively correlated to both patient survival and pathological complete response after neoadjuvant chemotherapy [2–5]. In addition, TILs have shown a predictive value in patients with TNBC who were treated with ICI monotherapy, and their assessment is being implemented as a stratification factor in breast cancer immunotherapy trials [20]. As previously described, CD8^+^ TILs (together with PD-L1 expression on immune cells) have been associated with increased PFS and OS in patients treated with atezolizumab and nab-paclitaxel in the IMpassion130 trial [18]. Conversely, stromal TILs were only able to predict PFS benefit. In this context, interesting findings have been provided by preliminary analyses of the KEYNOTE-173 trial [21], which is investigating the combination of pembrolizumab and chemotherapy in the neoadjuvant setting of TNBC. A recent exploratory analysis of this trial showed that high levels of pretreatment stromal TILs and PD-L1 expression, reported as a combined positive score, were significantly associated with higher pathologic complete response and overall response rates in TNBC patients treated with an immunotherapy-based combination [21].

Moreover, recent evidence has suggested that qualitative differences in a TIL subpopulation can better define patient prognosis [22]. CD8^+^ T cells with features of tissue-resident memory T cell differentiation were described in the lymphocytic infiltrate from breast tumors; the CD8^+^ tissue-resident memory gene signature subsequently developed was shown to be significantly associated with improved patient survival in early-stage TNBC [22].

### Gene signatures

In conjunction with TILs, multiple gene signatures have been studied as surrogates of breast cancer immunogenicity. A recent proposal classified breast cancer into four categories (immunologic constants of rejection (ICR) ICR1 through ICR4) according to their immune-related gene expressions, with these categories being correlated with survival in a retrospective in silico simulation [23]. Specifically, the T helper 1 phenotype (ICR4), associated with an upregulation of immunoregulatory transcripts such as PD-L1, PD-1, FOXP3, IDO1, and CTLA-4, was correlated with a prolonged patient survival. Conversely, the presence of MAPK pathway disruptions was tightly associated with an immune-unfavorable phenotype (ICR1), suggesting that alterations in this pathway are linked to a negative regulation of immune response in breast cancer. Interestingly, inhibition of MEK, a crucial molecule of the MAPK pathway, was able to increase PD-L1 and MHC class I expression on TNBC cells, synergizing with PD-L1/PD-1 inhibition in inducing antitumor immune responses in TNBC mouse models [24]. In a further study, a four-gene signature (HLF, CXCL13, SULT1E1, and GBP1) was found to predict an increased number of TILs and an improved disease-free survival in early stage TNBC [25]. However, these gene signatures have not yet been tested in metastatic TNBC patients and their role in predicting response to ICIs remains to be defined.

### Tumor mutational burden (TMB)

A high TMB has been associated with immunogenicity in several tumor types [26] and correlated with clinical response and increased survival after ICI-based immunotherapy in patients with melanoma, lung, and colorectal cancers [27–30]. TMB is a measurement of the number of nonsynonymous mutations carried by tumor cells [27]. Mutations lead to increased expression of neoantigens in the context of MHC class I antigens, enhancing the recognition of cancer cells by T cells. However, limited data regarding TMB in breast cancer is available. From genomic data, patients with a favorable immune subclass (based on ‘positive’ immune-infiltrate disposition) along with a high TMB have a better prognosis [31]. In addition, a higher TMB is more frequent in TNBC as compared to hormone receptor-positive subtypes [31]. In contrast with these findings, Samstein et al. [30] recently published a wide analysis of clinical and genomic data from more than 1600 advanced cancer patients treated with ICIs, and reported no significant differences for breast cancer patients in terms of survival after immunotherapy treatment. In order to reconcile these discrepancies, we presume that a high TMB alone does not represent the optimal predictor for immunotherapeutic response in breast cancer, suggesting that a finer selection to enrich the TNBC patient population is required.

### MSI and MMR deficiency

Microsatellites are tandem repeats of short DNA sequences, abundant throughout the human genome. MSI is a hypermutator phenotype that occurs in some tumors with an impaired DNA MMR [32]. MMR deficiency is known to occur in some tumors, either by somatic hypermutation of MMR genes, an inherited germline MMR pathway mutation, or double somatic mutations in MMR genes [33]. Recently, tumors harboring a high MSI have been found to be susceptible to ICI-based immunotherapy [34, 35], leading to the approval of the anti-PD-1 agent pembrolizumab for any high MSI or MMR-deficient unresectable or metastatic solid tumor. MSI incidence in breast cancer has not yet been fully elucidated, although high MSI in breast cancers seems to be found in less than 2% of cases [32]. In a large analysis of more than 1900 breast cancers [36], high-MSI tumors presented a low incidence (0.6%); however, a conjoined analysis of PD-L1 expression, high TMB, and high MSI selected up to 13% of TNBC patients with at least one of these alterations. Considering that approximately 5% of unselected patients with breast cancer carry a germline BRCA mutation [37], BRCA1 mutations are predisposed to TNBC, being discovered in 40–50% of cases [38]. Given the central role of BRCA1 in homologous recombination-mediated DNA repair [39], BRCA1-mutated TNBC showed a higher somatic mutational load, a greater number of TILs, and an increased expression of immunomodulatory genes (PD-1 and CTLA-4) as compared to BRCA1-wildtype TNBC [40]. Interestingly, the combination of two ICIs (against PD-1 and CTLA-4, respectively) with cisplatin treatment attenuated growth and improved survival in an in vivo BRCA1-deficient TNBC model, providing a rationale to implement immunotherapeutic strategies in this subgroup of TNBC. Several clinical trials are testing a combination of ICIs and PARP inhibitors (e.g., olaparib, niraparib, and talazoparib) with preliminary data of activity [41, 42].

## Choosing the right chemotherapeutic partner for immunotherapy

Despite assessments regarding the ideal chemotherapeutic partner for combination treatment with ICIs, various questions remain. Nab-paclitaxel was initially selected in the IMpassion130 study because it facilitates the reduced use of corticosteroids [43]. However, better agents may be available to enhance immunogenicity of breast cancer, including anthracyclines, platinum salts, and other taxanes [44]. Chemotherapy can induce multiple immunomodulatory changes in the tumor microenvironment, including increased antigen release by tumor cells, PD-L1 upregulation, and hyperexpression of immunogenic cell surface markers (e.g., MHC class I). Collectively, these modifications might positively influence the effectiveness of immunotherapy [45, 46]. Specifically, various chemotherapeutic drugs routinely adopted for TNBC treatment can induce distinct effects on the immune system, as described in detail below.

### Anthracyclines

Anthracyclines are capable of inducing immunogenic cell death (ICD), a form of apoptosis which can induce an effective antitumor immune response through activation of dendritic cells and specific T cell response [47]. In addition, anthracyclines can also increase proliferation of CD8^+^ T cells.

### Taxanes

Taxanes can increase TIL recruitment in primary breast cancer [48]. Moreover, taxanes have been shown to selectively decrease T regulatory and myeloid-derived suppressor cells (MDSCs), partially reducing immunosuppression in the tumor microenvironment [49–51]. We have to highlight that these immunomodulating effects have been described for old generation taxanes (docetaxel and paclitaxel); no preclinical data regarding nab-paclitaxel’s activity on the immune system have been reported so far.

### Cyclophosphamide

Cyclophosphamide, in conjunction with its well-known ability to induce ICD, can suppress T regulatory cells and increase the proliferative capacity of CD8^+^ T cells and natural killer cells [52, 53].

### Gemcitabine

Gemcitabine can reduce MDSC number and increase the anti-tumor activity of CD8^+^ T cells [54, 55].

### Platinum salts

Platinum salts have been shown to induce ICD as well as to increase MHC class I complex on tumor cells [56, 57], also promoting T cell activation and downregulating MDSC function [58].

## Single agent versus combination immunotherapy in TNBC

In addition to the results of the IMpassion130 study [11], other clinical trials are currently evaluating chemotherapy and immunotherapy combinations in TNBC patients. Preliminary data are available for the open-label phase Ib/II KEYNOTE-150 trial assessing the combination of eribulin and pembrolizumab [59]. Among 107 metastatic TNBC patients (106 evaluable for efficacy), 65 were treatment naïve, while 41 had received one to two prior lines of therapy. Half of the patients in the study had PD-L1^+^ TNBC (45.8%). The ORR of the combination treatment in the overall population and that in the untreated and pretreated patients was 26.4, 29.2, and 22.0%, respectively. Clinical activity was observed regardless of PD-L1 expression (ORR 30.6% for PD-L1^+^ (*n* = 49) and 22.4% PD-L1^−^ (*n* = 49)), even if a greater number of responses was reported in the PD-L1^+^ subgroup. The PFS and OS across the full study were 4.2 and 17.7 months, respectively. In the first-line and second−/third-line settings, the median PFS was 4.9 and 4.1 months, respectively, while the median OS was 17.7 and 18.3 months. Considering that KEYNOTE-150 was a single-arm phase Ib/II trial, the results are largely comparable with those obtained from the IMpassion130 study.

While the combinations of atezolizumab with nab-paclitaxel and pembrolizumab with eribulin produced substantial benefits in TNBC patients, we hypothesize that some subgroups of breast cancer patients (e.g., those with high TILs or high PD-L1 expression) may derive benefit from ICI monotherapy; evidence of this is available from phase I/II clinical trials (Table 2).

**Table 2:** Completed studies with immune checkpoint inhibitors in triple-negative breast cancer

Trial	Setting	Drug	Patients	Results	Ref.
Single agent immunotherapy					
KEYNOTE-012 NCT01848834	Advanced PD-L1^+^ TNBC	Pembrolizumab 10 mg/kg Q2W	27	ORR, 18.5% Median PFS, 1.9 months Median OS, 11.2 months	[60]
KEYNOTE-086 NCT02447003	Advanced, untreated, any PD-L1 TNBC (cohort A) Advanced, untreated PD-L1^+^ TNBC (cohort B)	Pembrolizumab 200 mg Q3W	Cohort A: 170 Cohort B: 84	ORR Cohort A, 4.7% ORR Cohort B, 22.6% Median PFS Cohort A, 2 months Median PFS Cohort B, 2.1 months Median OS Cohort A, 8.9 months Median OS Cohort B, 19.2 months	[20, 61]
JAVELIN NCT01772004	TNBC unselected for PD-L1 (68.8% had PD-L1^+^ tumors)	Avelumab 10 mg/kg every 2 weeks	58	ORR, 5.2% (22.2% in PD-L1^+^) Median PFS, 1.5 months Median OS, 9.2 months	[62]
NCT01375842	Advanced TNBC unselected for PD-L1 (65.7% had PD-L1^+^ tumors)	Atezolizumab 15 or 20 mg/kg, or at a 1200-mg flat dose, Q3W	116	ORR, 10% (12.7% in PD-L1^+^) Median PFS, 1.4 months by RECIST Median PFS, 1.9 months by irRECIST Median OS, 8.9 months	[63]
Combination of immunotherapy and chemotherapy					
KEYNOTE-150 NCT02513472	Advanced TNBC unselected for PD-L1	Eribulin ± pembrolizumab 200 mg Q3W	107	ORR, 26.4% (30.6% in PD-L1^+^) Median PFS, 4.2 months Median OS, 17.7 months	[59]
IMpassion130 NCT02425891	Untreated metastatic TNBC unselected for PD-L1	Nab-paclitaxel ± atezolizumab 840 mg Q2W	902 (451 treated with atezolizumab)	ORR, 56% (58.9% in PD-L1^+^) Median PFS, 7.2 months Median PFS, 7.5 months (PD-L1^+^) Median OS, 21.3 months Median OS, 25 months (PD-L1^+^)	[11, 64]

PD-L1^+^ expression in stroma or ≥ 1% of tumor cells by immunohistochemistry

*irRECIST* immune-related Response Evaluation Criteria In Solid Tumors, *ORR* objective response rate, *OS* overall survival, *PD-L1* programmed death-ligand 1, *PFS* progression-free survival, *Q2W* every 2 weeks, *Q3W* every 3 weeks, *RECIST* Response Evaluation Criteria In Solid Tumors, *TNBC* triple-negative breast cancer

In a phase I clinical trial [63], atezolizumab led to a higher ORR in the first-line setting (24%) compared to a second-line or greater setting (6%). In first-line patients, median OS was 17.6 months. Interestingly, patients with PD-L1 expression in at least 1% of tumor-infiltrating immune cells had higher ORRs (12 vs. 0%) and a longer OS (10.1 vs. 6.0 months) than those with PD-L1 expression in less than 1% of tumor-infiltrating immune cells. High levels of immune cells (> 10%) were independently associated with higher ORR and longer OS.

In the phase Ib KEYNOTE-012 trial [60], the anti-PD-1 agent pembrolizumab achieved an ORR of 18.5% in metastatic TNBC patients, with a median OS of 11.2 months. Of note, 15.6% of the subjects enrolled in this trial were treatment naïve. Similarly, in the phase II trial KEYNOTE-086 (cohort-A) [61], pembrolizumab led to an ORR and disease control rate of 5.3 and 7.6%, respectively, in pretreated TNBC patients; median PFS and OS were 2.0 and 9.0 months, respectively. Patients with PD-L1^+^ tumors showed only a slight increase in response rate. Additionally, approximately 40% of patients received more than three lines of therapy for metastatic disease.

Finally, in the phase I JAVELIN trial [62], the anti-PD-L1 agent avelumab produced an ORR of 5.2% in heavily pretreated metastatic TNBC patients. A trend toward a higher ORR was seen in patients with PD-L1^+^ versus PD-L1^−^ tumor-associated immune cells in the overall population (16.7% vs. 1.6%) and in the TNBC subgroup (22.2% vs. 2.6%).

As expected, a high response to single-agent anti-PD-1/PD-L1 immunotherapy has been observed in previously untreated metastatic TNBC patients. These findings suggest that ICIs should be more active in less heavily pretreated patients, opening the door to testing these strategies in an early TNBC setting.

## Moving immunotherapy to early TNBC

Previous evidence suggests that early TNBC presents with a reduced immunosuppressive phenotype compared to metastatic cases [65]. Therefore, there is increasing interest in testing immunotherapeutic strategies in both neoadjuvant and adjuvant settings. Data is available on the efficacy of ICIs in early settings obtained in stage III melanoma and non-small cell lung cancer [66–68]. In TNBC, various neoadjuvant studies are currently ongoing (Table 1).

Neoadjuvant trials represent an excellent in vivo laboratory to test immunotherapeutic agents and their potential combination with other drugs, including chemotherapy, targeted agents, and other immunomodulatory agents. The possibility to obtain baseline biopsies and to reassess both tumor response and changes in the tumor microenvironment at established time-points can lead to the discovery of novel biomarkers for patient stratification. Innovative findings obtained in the neoadjuvant setting could then be translated in adjuvant and metastatic settings. However, an additional important point of discussion in the neoadjuvant setting should be raised – when defining endpoints for immunotherapy studies, should OS and event-free survival be preferred instead of pathologic complete response? In other solid tumors, the real benefit of ICI administration is represented by an increase in OS, and therefore pathologic complete response might not be the ideal surrogate endpoint to approve these agents in the neoadjuvant setting. The selection of adequate endpoints is strongly encouraged for future clinical trials testing immunotherapy in TNBC.

Regarding the adjuvant setting, TNBC patients at high risk for relapse and who are less likely to be cured by the current standard of treatment could benefit the most by the addition of ICIs. For instance, patients with TNBC who do not achieve pathologic complete response after neoadjuvant chemotherapy have a worse prognosis, and capecitabine administration in the post-neoadjuvant setting is the only standard of care for these patients [69]. The addition of ICIs could improve cure rates in this setting; some trials are exploring this possibility (Table 1).

## Conclusion

The harnessing in clinical practice of immune checkpoint-based treatment has radically changed the therapeutic approaches for several tumor types. Nevertheless, until the IMpassion130 trial, few studies had addressed immunotherapeutic strategies for the treatment of breast cancer. The IMpassion130 trial first explored the metastatic setting, with lessons being learned primarily from phase I trials. First, ICIs should be combined with other agents to improve benefit. Second, immunotherapy should be implemented in the first-line setting of metastatic treatment to improve response rates. Third, patients should be stratified according to specific biomarkers. Additionally, early-stage breast cancer appears to be even more appealing than the metastatic setting for the introduction of ICIs, both in the neoadjuvant and adjuvant settings, since primary tumors seem more immunogenic than metastatic sites. The multiple ongoing trials may shed light on breast cancer immune response biomarkers and help determine whether a multidimensional immunogram could predict efficacy better than the current PD-L1-based unidimensional immunogram.

## Abbreviations

ICD: immunogenic cell death
ICIs: immune checkpoint inhibitors
ICR: immunologic constants of rejection
ITT: intent-to-treat
MDSCs: myeloid-derived suppressor cells
MMR: mismatch repair
MSI: microsatellite instability
OS: overall survival
PD-L1: programmed death-ligand 1
PFS: progression-free survival
TILs: tumor-infiltrating lymphocytes
TMB: tumor mutational burden
TNBC: triple-negative breast cancer
